# In-situ laboratory monitoring of cyanobacterial influence on calcite dissolution

**DOI:** 10.1038/s41529-025-00712-5

**Published:** 2025-11-22

**Authors:** Luca Stigliano, Bastien Wild, Karim Benzerara, Philippe Ackerer, Cynthia Travert, Kevin G. Knauss, Damien Daval

**Affiliations:** 1 Univ. Grenoble Alpes, Univ. Savoie Mont Blanc, CNRS, IRD, Univ. G. Eiffel, ISTerre, Grenoble, France; 2 Institut de Minéralogie, de Physique des Matériaux et de Cosmochimie (IMPMC), Sorbonne Université, Museum National d’Histoire Naturelle, UMR CNRS 7590, Paris, France; 3 ITES Institut Terre et Environnement de Strasbourg - CNRS/Université de Strasbourg, Strasbourg, France; 4 Energy Geosciences Division, Lawrence Berkeley National Laboratory, Berkeley, CA USA; 5 Present Address: Department of Earth Sciences, University of Oxford, Oxford, UK

**Keywords:** Biogeochemistry, Ecology, Ecology, Environmental sciences, Microbiology, Solid Earth sciences

## Abstract

Microbial interactions with mineral surfaces play a critical role in biogeochemical cycles, yet their dynamic coupling with mineral reactivity remains poorly constrained. Here, in-situ time-resolved monitoring of topographic evolution of the calcite-bacteria interface was performed using a fluid cell coupled to vertical scanning interferometry (VSI). The cyanobacterial strain *Chroococcidiopsis thermalis* PCC 7203 was inoculated onto polished and pre-etched calcite surfaces under conditions strongly undersaturated or closer to calcite saturation. The formation of localized topographic highs, produced by dissolution of surrounding material, was found to correlate with the residence time of attached cells at *Ω* = 0.0, but not at Ω = 0.3. Physiological tests suggested that the composition of the bulk fluid modulates microbial activity, thereby influencing interfacial pH, and in turn, calcite reactivity. Moreover, calcite reactivity was found to exert a stronger control on bacterial detachment dynamics than initial surface roughness or surface charge under the tested conditions. These findings emphasize the importance of microscale feedbacks between microbial colonization and mineral weathering, and demonstrate the potential of in-situ interferometric imaging for probing the dynamics of processes at microbe-mineral interfaces.

## Introduction

The vast majority of microbes on Earth live attached to solid substrates. Depending on the considered studies, the ratio of attached to unattached cells ranges between 100:1 and 1000:1^1–3^, and a significant proportion of sessile cells (between 20 and 80%) are estimated to exist as biofilms^4^. The benefits associated with cell adhesion to solid substrates are manifold, and include (i) water retention and protection against dehydration^4^; (ii) the development of a microenvironment in which the concentration of key nutrients is increased^5^, and where the local conditions are overall decoupled from ambient environmental conditions; and (iii) potential direct access to an energy source, as some mineral surfaces can represent electron acceptors for a number of microorganisms such as iron-reducing bacteria^6^.

Because of the central role of microbe-mineral contacts in Earth sciences, as well as for several issues related to medicine (e.g.,^7^ and references therein) and civil engineering (e.g.,^8^ and references therein), a substantial literature has been dedicated to elucidating the parameters that control the adhesion of microorganisms to inert materials and/or under conditions where the substrate is poorly reactive. Several studies have demonstrated that substrate surface properties such as chemical composition^9^, hydrophobicity^10^, free energy^11^ and roughness^12,13^, but also patterning of the microtopography^14^, all contribute to modifying the rates and mechanisms of bacterial attachment.

Microbial attachment is often considered as the first step towards the development of microcolonies and eventually, biofilms, and sometimes considered as a pre-requisite for initiating interactions with the considered substrate. In fact, while microorganisms are known to secrete a range of dissolved species that enhance the dissolution rate of minerals (e.g.,^15^, and references therein), the concentrations required to significantly increase the reactivity of minerals are not typically reached in bulk environmental fluids and/or soil solutions (e.g.,^16^). Conversely, microenvironments developed at the microbe-mineral interface, within microcolonies and/or biofilms may sustain conditions that promote enhanced dissolution fluxes^17,18^.

Many in vitro experiments have demonstrated that bacteria can promote mineral weathering (e.g.,^19–23^), but whether this enhancement is localized at the microbe-mineral interface and actually results from the development of microenvironments in the vicinity of the microbe-mineral contact remains controversial. Foundational studies on boring cyanobacteria have long demonstrated their capacity to colonize lithic substrates and drive bioerosion, both through direct boring activity and indirect chemical alteration of the mineral matrix^24,25^. More recently, physiological investigations have also revealed how endolithic cyanobacteria adapt to extreme environments and modulate oxidative stress during mineral colonization^26^. Beyond cyanobacteria, Ahmed et al.^27^ showed that the attachment of six different microbial species isolated from a podzol soil to biotite resulted in a greater dissolution rate of the substrate compared with similar experiments conducted with unattached cells. Conversely, Perez et al.^28^ reported that *Pseudomonas aeruginosa* more efficiently enhanced the dissolution of Fe(III)-bearing glass when experiments were conducted with unattached cells. These seemingly contrasting findings are likely not incompatible, but instead illustrate that the impact of cell attachment may be substrate- and/or species-dependent. Furthermore, and perhaps more surprisingly, other studies have suggested that both local enhancement and global inhibition of dissolution can occur simultaneously. Davis et al.^29^ and Davis et al.^30^ showed that *Shewanella oneidensis* MR-1 attachment to calcite surfaces led to significant localized dissolution (referred to as “microbial entrenchment” by the authors), while the overall calcite dissolution flux was higher in abiotic than in biotic experiments. They hypothesized that the surface dynamics (in particular, surface retreat) represented a significant obstacle to cell attachment. Consequently, when a given cell successfully attached to high-energy sites such as those from which etch pits emanate, this attachment contributed to preventing the formation of etch pits and resulting stepwaves, ultimately decreasing calcite dissolution rates and, therefore, increasing colonization of the surface by *Shewanella* species. Thus, although the attachment of *Shewanella* sp. to calcite surface locally enhanced dissolution (bacterial entrenchment), this effect was globally outweighed by the dissolution-inhibiting impact of suppressed etch pits nucleation, which is the primary driver of mineral surface retreat.

Altogether, these results suggest that surface dynamics may represent a supplementary controlling-factor of microbial attachment to mineral surface, and vice versa. However, unlike inert materials for which the interactions between microbes and substrate can be satisfactorily investigated by observing the final bacterial coverage of a given substrate under given experimental conditions, such an approach insufficiently captures the relations between microbial attachment, surface reactivity, and their mutual feedbacks, as these processes are intrinsically dynamic and intertwined. Their investigation would therefore greatly benefit from real-time in situ observations.

In a previous study^31^, we used the cyanobacterial strain *Chroococcidiopsis thermalis* PCC 7203, particularly targeted as a model strain in astrobiology^32^ to test whether the surface of calcite imprinted a specific microtopographic signature resulting from its interaction with bacteria. In the presence of bacteria and under conditions favoring spontaneous nucleation of etch pits (commonly referred to as “far-from-equilibrium conditions”; e.g.,^33^^,^^34^), observation of calcite surface microtopography after a few days of incubation exhibited statistically more abundant high-elevation regions, making microbially-weathered surfaces quantitatively distinguishable from their abiotic counterparts. However, the dynamics of formation of such high-elevation regions remained out-of-reach of this previous study, as observations of the final surface required the physical63 removal of attached cells, thus marking the end of a given experiment, providing only a single “snapshot” of the dissolution process. Consequently, the impact of several parameters such as the residence time of bacteria at the calcite surface, the ongoing microtopography evolution of the substrate, and the link between surface reactivity and microbial attachment/detachment rate, remained unknown. Accessing this information is critical for understanding the mechanisms of formation of high-elevation regions, which we previously suggested to result from the local microenvironment at the cyanobacteria-calcite interface, where the solution was closer to calcite saturation. Moreover, a refined understanding of the mechanisms controlling mineral reactivity at the microbe-mineral interface may help to assess the potential of using surface roughness as a relevant parameter for defining imprints of past microbe-mineral interactions.

To this end, here we performed in situ and real-time monitoring of the evolution of the microtopography of calcite surfaces inoculated with *C. thermalis* PCC 7203. Initial calcite surfaces were used either polished (“untreated”) or “pre-etched” in deionized water to generate contrasted initial surface roughness, thereby disentangling the role of surface reactivity from that of the fluid composition. Their evolution in contact with cyanobacteria was studied under solutions with different saturation indexes with respect to calcite. We recall that the saturation index (*Ω*) is defined as the ratio of the ion activity product to the solubility product (Ksp), where *Ω* < 1 indicates undersaturation and favors dissolution. This experimental design was intended to generate separate statistical differentiations of biotically weathered calcite, expected to be more pronounced at lower *Ω* values, corresponding to far-from-equilibrium conditions^31^.

## Results

### Calcite reactivity

Calcite dissolution rates were measured in three of the four experiments conducted in this study, based on the relative difference of elevation between the calcite crystal and an inert quartz reference crystal (see “Methods”). Three sub-windows were considered for each experiment to estimate the intrinsic variability in reactivity across the calcite surface.

**Fig. 1:**
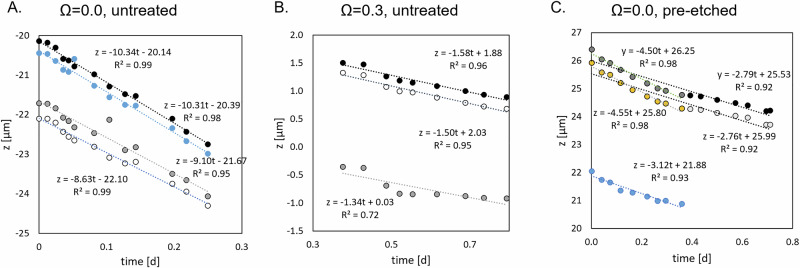
Calcite dissolution rates under varying saturation states and surface treatments. Calcite surface retreat, measured as the height difference between the inert quartz reference and the reactive calcite surface, is plotted as a function of time. **A** Untreated calcite at *Ω* = 0.0; **B** untreated calcite at *Ω* = 0.3; **C** pre-etched calcite at *Ω* = 0.0. Dotted lines show linear regressions, and their slopes represent the calcite dissolution rate for each condition.

As expected, the dissolution rate of calcite was higher at *Ω* = 0.0 than at *Ω* = 0.3. For the untreated surface, the dissolution rate of calcite ranged from 8.5 to 10.3 µm.d^–1^, depending on the considered sub-window (Fig. 1A). No significant evolution of the rate with time was observed over both short (~6 h) and long (up to ~24 h) durations. In comparison, the reactivity of the pre-etched surface over short durations (~6 h) was lower, ranging from 3.1 to 4.5 µm.d^–^^1^ depending on the considered sub-window. Of note, a slight decrease was observed over longer durations (up to ~18 h), with the dissolution rate dropping to ~2.7 µm.d^–^^1^ (Fig. 1B). Finally, the lowest dissolution rates were measured for the untreated calcite at *Ω* = 0.3, ranging from 1.3 to 1.6 µm.d^–^^1^ over ~5 h (Fig. 1C).

### Dynamics of cell detachment

The initial characteristics of the cell coverage on calcite and quartz surfaces (namely surface density, spatial extent and thickness of individual clusters) showed no apparent correlation with either the fluid composition or the initial topography of the calcite substrates. Within each experiment, regions of higher cell cluster density were regularly detected alongside areas with sparse or no detectable cell coverage. Similarly, the properties of individual cell clusters (i.e., morphology, thickness, lateral extent, number of bacterial cells) attached to the surfaces of both calcite and quartz were highly variable, ranging from cell monolayers to dense aggregates exceeding tens of microns in thickness and lateral extent. Due to limited experimental control on the initial distribution and properties of cell patches deposited on both calcite and quartz surfaces during cyanobacteria injection (see “Methods”), we chose to focus on the dynamics of cell detachment by maintaining the properties of cell coverage as comparable as possible from one experiment to the next. Surfaces involving monolayers of cells attached to the surface were by far the configurations enabling the quantification of cell detachment events with the highest degree of confidence. Accordingly, the dynamics of cell detachment quantified in the present refer to the detachment of individual cells from monolayer configurations. A movie representing the evolution of a representative region of interest where cell detachment was quantified (Experiment #1, *Ω* = 0.0, untreated calcite surface) is provided in the Supplementary materials.

**Fig. 2:**
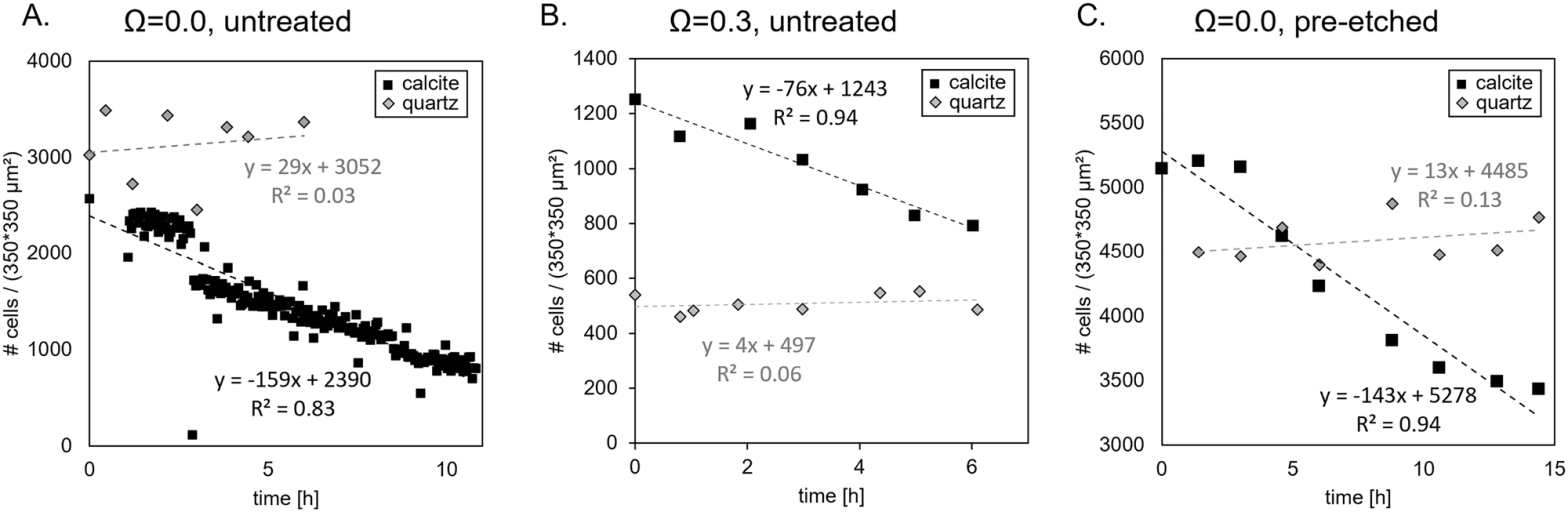
Bacterial detachment rates from calcite and quartz surfaces under varying saturation states and surface treatments. Bacterial coverage of calcite and quartz surfaces is plotted over time for **A** untreated calcite at *Ω* = 0.0, **B** untreated calcite at *Ω* = 0.3, and **C** pre-etched calcite at *Ω* = 0.0. Dotted lines represent linear regressions, with slopes corresponding to the bacterial detachment rate for each condition.

On quartz samples, no statistically significant cell detachment was detected. Variations in the estimated number of cells between two timesteps were thus interpreted as reflecting the uncertainties in data processing, which was in the order of ±8% across the three experiments encompassing a quartz crystal (Fig. 2).

Conversely, the detachment of cells from the calcite surface was consistently observed and quantified across all experiments, irrespective of the fluid composition and/or the initial roughness of the substrate (note that it was not possible to estimate uncertainties associated with image processing in the same way as for quartz because detachment was continuous on calcite). Overall, the detachment rate ranged between 620 and 1298 cells.mm^–2^.h^–1^. The detachment rate was found to be similar for experiments conducted at *Ω* = 0.0 (1167 vs. 1298 cells.mm^–2^.h^–1^ for experiments conducted with pre-etched and untreated surfaces, respectively) and significantly lower in Experiment #2 (*Ω* = 0.3, untreated calcite surface). Visual inspection further suggested a close association between cell detachment and the propagation of steps originating from pre-existing etch pits, bearing high step density.

### Evolution of calcite surface topography

Calcite surface roughness was evaluated in all four experiments using the arithmetic mean roughness (*R*_*a*_), calculated as:

1 $${R}_{a}=\frac{1}{N}\mathop{\sum }\limits_{i=1}^{N}\left|{z}_{i}-{\boldsymbol{z}}\right|$$

where *z*_*i*_ is the surface height at a given pixel and ***z*** is the mean height of the measured area. For each experiment, three sub-windows of 30 × 30 µm^2^ were selected across the calcite surfaces to account for spatial heterogeneity. Surface microtopography data were acquired using vertical scanning interferometry (VSI).

In experiments with untreated calcite surfaces, the final roughness *R*_*a*_ values were measured to be 41.3 ± 1.0 nm at *Ω* = 0.00 (Experiment #1) and 12.3 ± 0.8 nm at Ω = 0.30 (Experiment #2). For the pre-etched surfaces, final roughness values were slightly higher under both conditions, with *R*_*a*_ = 47.1 ± 4.8 nm at *Ω* = 0.00 (Experiment #4) and *R*_*a*_ = 20.8 ± 3.6 nm at *Ω* = 0.30 (Experiment #3). These observations are consistent with our previous findings^35^, which demonstrated an inverse relationship between steady-state surface roughness and saturation state, whereby lower *Ω* values result in higher roughness.

For comparison, *R*_*a*_ was also measured for both *Ω* conditions on the surface of the inert quartz reference sample, which was placed adjacent to the calcite chip. Across all conditions, the roughness of quartz remained stable throughout the experiments, with *R*_*a*_ = 1.5 ± 0.1 nm.

Moreover, for each experiment, “bactFeria residence time maps” were generated by tracking the cumulative duration of bacterial coverage for each pixel over time-resolved VSI data. These maps provide a spatial representation of how long each surface region was occupied by cells during the course of the experiments.

**Fig. 3:**
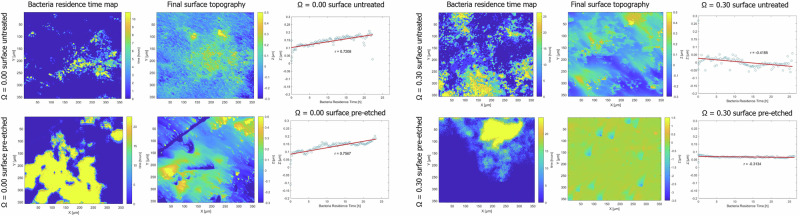
Correlation between bacteria residence time and final calcite surface topography for all experimental conditions (untreated calcite at *Ω* = 0.0 and *Ω* = 0.3; pre-etched calcite at *Ω* = 0.0 and *Ω* = 0.3). For each condition, panels show the bacteria residence time map (left), final VSI topography (middle), and pixel-wise correlation plot (right).

To determine whether bacterial presence affected the local evolution of surface topography, the final elevation (*z*-value) of each pixel was compared against its corresponding bacterial residence time. A pixel-wise correlation analysis revealed that at *Ω* = 0.00, regardless of surface pre-treatment (i.e., untreated vs. pre-etched), there was a positive correlation between residence time and surface height (*r* = 0.72 for untreated and *r* = 0.76 for pre-etched samples (Fig. 3)). This suggests that prolonged bacterial coverage locally inhibited dissolution, leading to the formation of topographic highs. In contrast, no such positive correlation was observed in the experiments conducted at *Ω* = 0.30 (*r* = –0.42 for untreated and *r* = –0.31 for pre-etched samples; Fig. 3), with both conditions exhibiting moderately negative correlations, suggesting that under these conditions, bacterial presence did not exert a measurable protective effect on the underlying calcite surface.

**Fig. 4:**
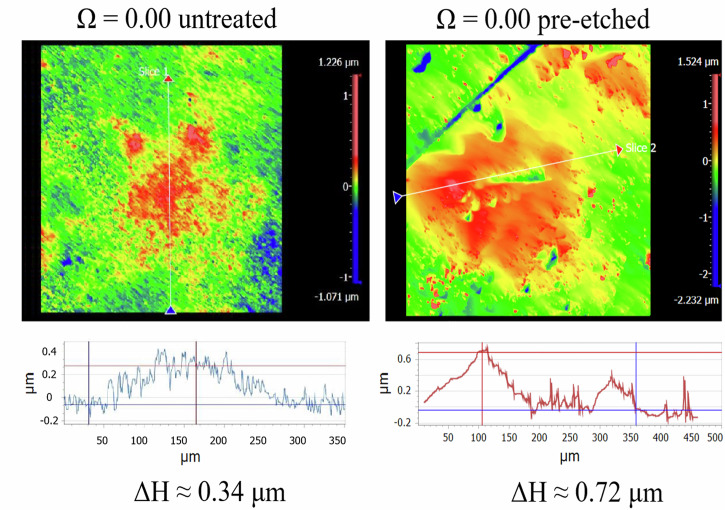
Final surface topography of calcite surfaces at *Ω* = 0.0 (untreated and pre-etched). Vertical Scanning Interferometry (VSI) images were acquired ex-situ at the end of the experiments after physical removal of bacteria. Height profiles show high-elevation regions formed beneath previously attached bacteria.

Lastly, microtopographic profile analysis revealed that at *Ω* = 0.00, prominent topographic highs developed during the experiments, reaching up to 0.34 µm in height for untreated surfaces and up to 0.72 µm for pre-etched surfaces (Fig. 4). No such features were identified under *Ω* = 0.30 conditions.

#### Ex situ physiological characterizations of *Chroococcidiopsis thermalis* PCC 7203

Since the *C. thermalis* PCC 7203 strain was inoculated into aqueous media that are suboptimal for its growth, basic physiological tests under continuous light exposure were performed to assess its tolerance to the experimental solutions.

In these tests, 3 mL of cell culture in a standard BG-11 growth medium (optical density: 4.175) were centrifuged for 10 min at 8000 rpm and resuspended in ~1 mL of either of the two solutions used in the flow cell experiments (i.e., either *Ω* = 0.0 or *Ω* = 0.3) before being added to a volume of ~120 mL of that same solution.

A first striking observation was the rise in solution pH measured within the first 24 h, which corresponds to the total duration of the in-situ experiments. A few minutes after incubation, pH increased from 7.92 ± 0.05 to 8.34 ± 0.05 and 8.10 ± 0.05 for the *Ω* = 0.0 and *Ω* = 0.3 solutions, respectively, reaching 9.28 ± 0.05 and 9.15 ± 0.05 after 24 h. Over the following 2 days, pH continued to increase, reaching 9.62 ± 0.05 and 9.75 ± 0.05 in the *Ω* = 0.0 and *Ω* = 0.3 solutions, respectively.

**Fig. 5:**
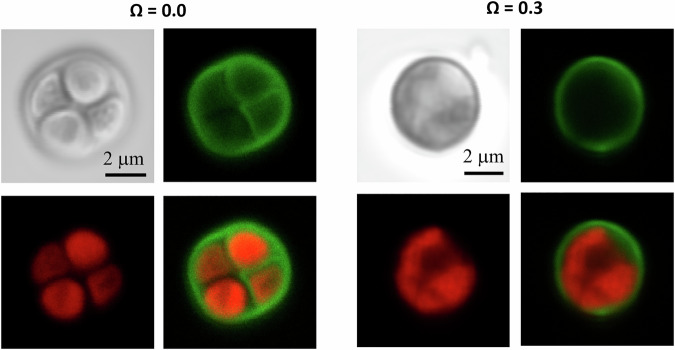
Confocal microscopy images of *Chroococcidiopsis thermalis* cells acquired after 72 h in *Ω* = 0.0 and *Ω* = 0.3 experimental fluids. Each panel shows phase contrast (top left), green fluorescence (cell envelope or EPS), red fluorescence (chlorophyll a autofluorescence), and merged images. Both conditions show preserved pigment fluorescence and envelope integrity, suggesting viability and metabolic activity under suboptimal conditions.

Fluorescence microscopy was performed after 3 days of incubation to assess the viability of *C. thermalis* PCC 7203 in the experimental solutions (see “Methods”). Epifluorescence imaging revealed a strong red fluorescence signal originating from within the cells (Fig. 5), characteristic of chlorophyll *a* and phycocyanin autofluorescence^36^. These observations (fluorescence and pH increase) indicate that the photosynthetic pigments were preserved, and suggest that the cells remained viable and metabolically active despite the suboptimal growth conditions, as further reflected by the observation of dividing cells in both solutions (Fig. 5). In addition, a green fluorescence signal outlining the cells was detected in most cases, which corresponds to the presence of intact cell envelopes and/or possibly extracellular polymeric substances (EPS) common in cyanobacteria exposed to stress. Taken together, these results support that *C. thermalis* cells tolerated the experimental conditions over the incubation period, maintaining structural integrity, pigment fluorescence and their positive impact on pH typical of physiologically functional cells.

## Discussion

Estimating mineral reactivity as promoted or inhibited by microbial activity through changes in fluid chemistry has long posed a significant experimental challenge. This is due to several factors, such as the difficulty of detecting the release of key cations in solutions with high background concentration (which is typically the case for experiments with *Ω* > 0), or achieving reliable mass balance while cations may be trapped in biofilms and/or microbial cells (e.g.,^37–40^). Complementary approaches include the quantification of cation depletion profiles at the surface of the altered substrate using techniques such as transmission electron microscopy (e.g.,^41^^,^^42^). However, while such methods are particularly suited for probing incongruent dissolution, they fail to capture the contribution of congruent dissolution to the overall flux. To overcome these limitations, recent studies have employed surface retreat measurements relative to an inert reference, which have proven effective in quantifying bioweathering rates, especially for minerals where incongruent dissolution is negligible (e.g.,^31^^,^^43^). To the best of our knowledge, the present study is the first to apply this approach to probe mineral bioweathering rate in situ and in real time.

At *Ω* = 0.0, calcite dissolution rate ranged from 8.5 to 10.3 µm.d^–1^ for the untreated surface, and from 2.7 to 4.5 µm.d^–1^ for pre-etched surface. Using the exact same fluid composition, Bouissonnié et al.^34^ reported abiotic dissolution rates for untreated calcite surface ranging from 1.4 to 1.7 µm.day^–1^ over 1-week experiments. Stigliano et al.^31^ reported that, under biotic conditions with *C. thermalis*, calcite dissolution rates could reach up to ~5 µm.d^–1^ over 5 days of reaction under similar conditions. While this latter value is in reasonable agreement with the estimates from the present study for pre-etched surface, it remains significantly lower than the range reported here for untreated surface (8.5 to 10.3 µm.d^–1^). A similar conclusion holds for the experiment conducted at *Ω* = 0.3, where calcite dissolution rate ranged from 1.3 to 1.6 µm.d^–1^, whereas Bouissonnié et al.^34^ reported abiotic dissolution rates ranging from 0.3 to 1.1 µm.d^–1^ for 0.2 < *Ω* < 0.4 over 1-week experiments. At *Ω* = 0.3, the dissolution rates reported by Stigliano et al.^31^ in the presence of *C. thermalis* did not indicate any enhancement (0.4 µm.d^–1^ at *Ω* ~ 0.3), and were consistent with abiotic rates reported by Boussonnié et al.^34^. Therefore, the rates determined in the present study are close to, but systematically higher than those reported in previous studies, by a factor of 2 to 4. Several explanations can account for those discrepancies:

(i) Intrinsic variability in crystal reactivity, which often exceed both analytical uncertainties and those arising from minor fluctuations in experimental conditions^44^ and can reach up to two orders of magnitude for a given protocol or fluid compositions^33,45^.

(ii) The intrinsic decline of mineral weathering rates over time (ageing effect) reported from laboratory experiments^46–48^ to field-scale estimates (e.g.,^49–51^), resulting from gradual depletion of energetically favorable surface sites^47^ as well as the progressive stabilization of poorly reactive crystallographic planes (e.g.,^52^). Supporting this hypothesis, dissolution rates for the pre-etched (i.e., aged) calcite surface in the *Ω* = 0.0 experiment were up to two times lower than those of the untreated surface in the present study.

(iii) The associated nature of transient initial high reactivity, as demonstrated in e.g., Smith et al.^53^, where the reactivity of the (104) plane of calcite was significantly higher during the first two days of dissolution, under conditions comparable to those in the present study (*T* = 20 °C, atmospheric pCO_2_, *Ω* ≤ 0.2, and identical NaCl and NaHCO_3_ concentrations). Given that the present experiments were conducted over day-long durations, it is likely that the experiments captured essentially this early, transient period.

Considering these three aspects, the rate data derived from the present study are in reasonable agreement with those reported in previous studies, and are confidently used in the next section to explore the link between calcite reactivity and the dynamics of cell detachment.

Moving from mineral reactivity to microbial dynamics, the attachment of bacterial cells to mineral surfaces under static conditions is governed by a complex interplay of physicochemical factors. Among them, the nature of the substrate itself plays a central role, with surface charge, roughness, and hydrophilicity all influencing microbial adhesion^54^. Environmental parameters related to solution chemistry such as pH, ionic strength, and the presence of divalent cations also modulate cell-surface interactions, by altering electrostatic forces or promoting bridging effects (ref.^55^ and references therein). Finally, the hydrodynamic conditions (in particular, flow velocity and associated shear stress) can either promote selective attachment or trigger detachment, depending on their magnitude and direction.

In the present study, biological variability was minimized by using a single axenic bacterial strain across all experiments, which allowed us to focus on the physicochemical controls on attachment and detachment. Nonetheless, we note that different cyanobacterial strains may exhibit distinct detachment behaviors (e.g., due to differences in EPS production), and such responses could be further modulated in mixed phototrophic communities. Hydrodynamic conditions were also kept as constant as possible, with an identical flow rate maintained throughout, although minor differences in crystal size may have introduced minor local variations. As such, observed differences in detachment behaviors can be primarily attributed to the identity of the substrate (calcite vs. quartz), changes in solution chemistry (*Ω* = 0.0 vs. 0.3), and potential dynamic modifications of the mineral surface itself. Below we discuss how each of these parameters are likely to influence cell attachment/detachment rates.

To the best of our knowledge, the zeta-potential of the *C. thermalis* strain has not been reported in the literature. However, studies on other cyanobacterial strains consistently indicate that their surfaces are negatively charged at circum-neutral pH (e.g.,^56–58^), due to the presence of deprotonated functional groups (such as carboxyl, amino, and hydroxyl) on the outermost cell surface layer. Regarding the substrates, the point of zero charge (pH_PZC_) is ~3 for quartz^59^ and 8–9.5 for calcite^60^. Under the conditions investigated here, quartz surfaces should therefore carry a strong negative charge, while calcite surfaces are expected to be neutral to slightly positive. From an electrostatic standpoint, this would favor cyanobacterial attachment to calcite over quartz.

**Fig. 6:**
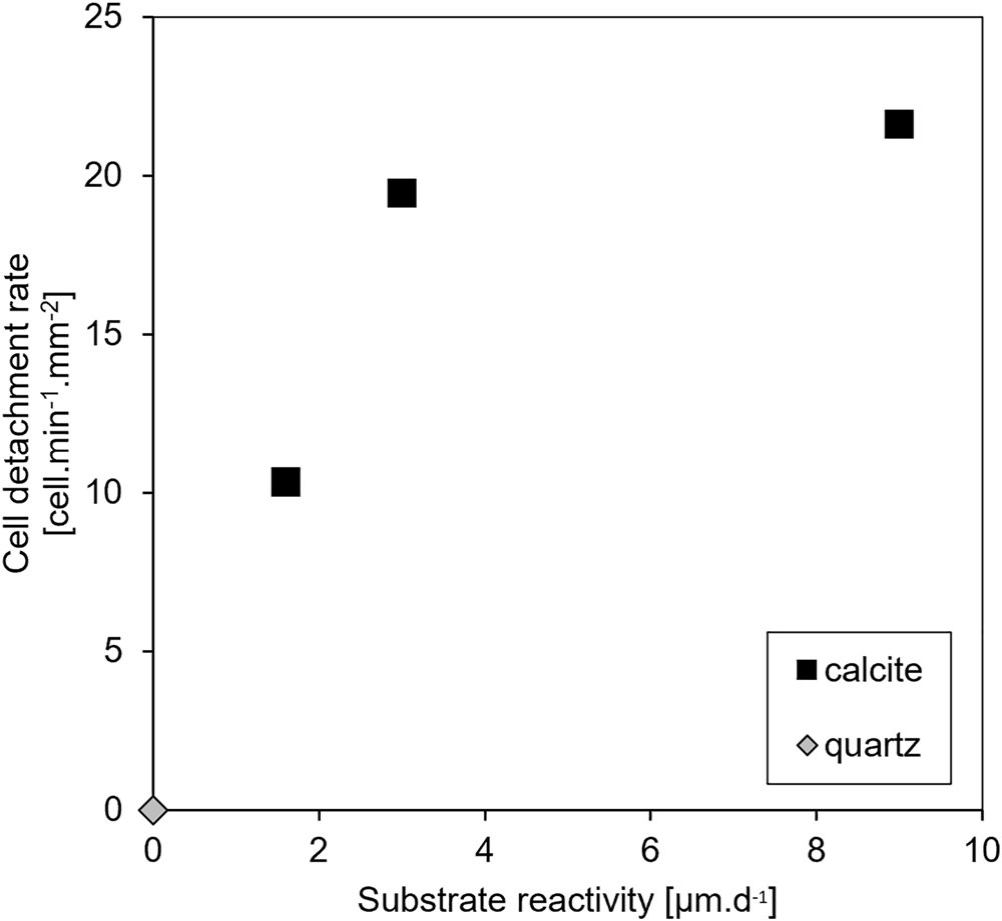
Relationship between bacterial detachment rates and calcite dissolution rates under varying saturation states and surface treatments. Bacterial detachment rates are plotted against the corresponding calcite dissolution rates under different experimental conditions (untreated calcite at *Ω* = 0.0; pre-etched calcite at *Ω* = 0.0; untreated calcite at *Ω* = 0.3). More reactive substrates exhibit higher detachment rates.

In terms of surface roughness, the following trend was observed (see Results): quartz < calcite (Experiment #2; *Ω* = 0.3, untreated surface) < calcite (Experiment #1; *Ω* = 0.0, untreated surface) < calcite (Experiment #4; *Ω* = 0.0, pre-etched surface). Again, this suggests that cyanobacteria should adhere more strongly to calcite than to quartz, with the lowest detachment rate expected for the pre-etched calcite surface (Experiment #4). However, this trend is not reflected in the actual detachment rates measured in the present study, which increases in the following order: quartz > calcite (Experiment #2) > calcite (Experiment #4) ~ calcite (Experiment #1). Notably, the fluid composition did not appear to influence the detachment of bacteria from quartz surface (compare the results of Experiments #1 and #4 (*Ω* = 0.0) to those from Experiment #2 (*Ω* = 0.3); see Fig. 2), leaving substrate reactivity as the most likely parameter controlling the detachment rate of bacteria from the mineral surfaces. Supporting this interpretation, the detachment rate of bacteria varies in line with the mineral dissolution rate (Fig. 6), assuming that quartz dissolution is negligible under the investigated conditions, which is a reasonable assumption, since quartz dissolution is known to be more than six orders of magnitude slower than calcite at circum-neutral pH (e.g.,^61^). Therefore, the present study suggests that in addition to previously identified factors influencing bacterial adhesion, the chemical reactivity of the substrate exerts a primary control on bacterial detachment rate, assuming that the bacterial activity was comparable across all experiments.

The present study also confirms earlier findings by Stigliano et al.^31^, showing that at the interface between attached microbial cells and the substrate, the primary effect of *C. thermalis* on calcite reactivity is a localized reduction in the dissolution rate at *Ω* = 0.0, while no significant effect is observed at *Ω* = 0.3. Supporting this conclusion, a positive correlation was observed between bacterial residence time (Fig. 3) and the development of topographic highs at *Ω* = 0.0, on both pre-etched and untreated calcite surfaces. In contrast, no such correlation was found in experiments conducted at *Ω* = 0.3.

Since calcite dissolution was confirmed in all experiments where a quartz reference was used, the formation of topographic highs at *Ω* = 0.0 most likely reflects local fluid compositions closer to calcite saturation at the bacteria-calcite interface. This localized increase in *Ω* is supported by the physiological tests reported earlier (see “Results”), which showed that even under nutrient-limited conditions, *C. thermalis* remained metabolically active and induced a rise in pH, likely due to its photosynthetic activity (e.g.,^62^).

The observed decrease in calcite reactivity under attached cells can be used to estimate local *Ω* values at the bacteria-calcite interface, assuming this decrease is primarily driven by an increase in pH. According to the empirical relationship between calcite dissolution rate and *Ω* proposed by Boussonnié et al.^34^, a reduction in calcite dissolution rate by up to 20% under attached cells (as observed in Experiment #4, Fig. 4) corresponds to a local increase in *Ω* from ~0.0 to 0.1.

This shift in *Ω* can in turn be used to infer local pH values, supposing that the increase in *Ω* mainly results from an increase in pH driven by metabolic activity of *C. thermalis*. The outlet steady-state calcium concentration ([Ca], in ppm) at *Ω* = 0.0 can be calculated from the following expression, which applies to flow-through systems (e.g.,^63^):

2 $$\left[{\rm{Ca}}\right]=\frac{r.{M}_{{Ca}}.{SA}}{{10}^{-6}.\nu }$$

where *M*_*Ca*_ is the molar mass of calcium (40 g.mol^–1^), *SA* is the calcite surface area (~10^–4 ^m^2^) and $\nu$ is the flow rate (0.004 mL/s). The calcite dissolution rate (*r*) at *Ω* = 0.0 is estimated to range between 9 × 10^–7 ^mol.m^–2^.s^–1^ (upper bound measured in the present study for Experiment #4) and 5 × 10^–7 ^mol.m^–2^.s^–1^ (lower bound, taken from Boussonnié et al.^34^). This range yields steady-state [Ca] values between 500 and 800 ppb in the flow cell.

Using [HCO_3_^–^] = 1 mmolal (matching the inlet stock solution) and a fixed pCO_2_ of 550 ppm (measured with a CO_2_ sensor in the experimental room), the local pH corresponding to *Ω* = 0.1 was computed using the CHESS code. The resulting pH at the bacteria–calcite interface ranges from 8.6 (for [Ca] = 800 ppb) to 8.7 (for [Ca] = 500 ppb), compared with a pH of 7.9 away from the cells.

If we now assume that the metabolic activity of the bacteria resulted in a similar local rise in pH in Experiment #2 (*Ω* = 0.3), then the resulting *Ω* at the calcite-bacteria interface would range from 6.0 to 9.3, i.e., well within the supersaturation domain for calcite. Therefore, under such conditions, the removal of microbial cells should have revealed even more pronounced topographic highs beneath attached cells. However, no height difference was observed over time between cell-covered and uncovered regions. Hence, this discrepancy suggests that the local pH increase at the bacteria-calcite interface was smaller in experiments conducted at *Ω* = 0.3 than at *Ω* = 0.0, implying a reduced metabolic (e.g., photosynthetic) activity of *C. thermalis* under these conditions. This interpretation is consistent with the results of the physiological tests, which revealed a more modest bulk pH increase within the first 24 h after incubation of the cells in the *Ω* = 0.3 solution than in the *Ω* = 0.0 solution (see “Results” section), despite identical exposure durations and nutrient conditions. Indeed, the absence of topographic highs for calcite surfaces reacted at *Ω* = 0.3 suggests that combined effects of metabolic activity (e.g., local pH shifts, diffusion changes, or EPS production) did not significantly alter the local saturation index at the bacteria-calcite interface, which remained close to that of the bulk solution.

Notably, the absence of topographic highs at *Ω* = 0.3 was consistent across both pre-etched and untreated calcite surfaces, which differ in surface reactivity. Since the solution chemistry was kept identical, the microbial metabolic response is expected to have been similar in both cases, implying that any surface patterning differences would therefore be attributed to differences in the surfaces’ sensitivity to local changes in dissolution rate. In general, pre-etched surfaces are expected to be more responsive to local changes in dissolution rates caused by shifts in saturation state. This expectation is confirmed by observations at *Ω* = 0.00, where, despite a lower global dissolution rate, the same microbial metabolic activity led to topographic highs that were approximately twice as prominent on pre-etched surfaces compared to untreated ones (see “Results” and Fig. 4). This difference likely arises from the higher density of dissolution stepwaves on pre-etched surfaces, which contain pre-existing etch pits. As shown in our earlier kMC modeling work, a greater number of stepwaves facilitates the development of more pronounced topographic features under conditions of locally reduced dissolution due to a local increase in saturation state^31^. Therefore, the consistent absence of topographic highs at *Ω* = 0.3 across both surface types further supports the interpretation that the local saturation index at the bacteria-calcite interface remained close to that of the bulk solution.

Altogether, these results suggest that the local inhibiting impact of *C. thermalis* on calcite dissolution, which is likely primarily driven by a local increase in pH at the cell–mineral interface, is most pronounced under far-from-equilibrium conditions (*Ω* ≈ 0), and becomes negligible at *Ω* ≥ 0.3. This interpretation is in line with earlier findings by Stigliano et al.^31^, which reported the presence of high-elevation regions resulting from locally inhibited dissolution only in *Ω* = 0.0 experiments, while no such features were observed at *Ω* = 0.3, 0.55, 0.6, or 0.8. While the inhibition of dissolution beneath attached cells can primarily be explained by localized pH elevation, EPS and other metabolic by-products may also contribute. The observation that the extent of inhibition varies with saturation state may further suggest that EPS production or properties respond to the degree of undersaturation, thereby influencing local mass transport and surface reactivity alongside pH effects. Taken together, these observations may point to the existence of a critical *Ω* threshold, above which the microbial modulation of surface reactivity becomes too weak to produce detectable topographic signatures over the timescale of the experiments. Nonetheless, determining the exact value of this saturation state threshold would require further investigation across a broader range of intermediate *Ω* values.

Altogether, the combination of in-situ VSI imaging and ex-situ physiological tests confirms that microenvironments developing at the microbe-mineral interface exert strong control on the local reactivity of the substrate. The novel approach introduced in the present study enables direct, time- and space-resolved quantification of dissolution dynamics, overcoming the limitations associated with traditional bulk measurements of microbially-mediated mineral weathering. It also offers a promising framework for identifying agnostic signatures of microbially-mediated mineral alteration that go beyond the conventional and often misleading criterion of topographic depression resembling bacteria in shape and size.

Crucially, this study establishes a novel link between bulk mineral reactivity and bacterial detachment dynamics under constant flow conditions, suggesting that less reactive surfaces allow for longer bacteria coverage, whereas more reactive surfaces are less prone to preserve bacteria coverage of the substrate. This insight opens new perspectives for understanding feedbacks between microbial colonization and surface transformation in diverse natural and engineered systems.

Beyond geochemical systems, such approaches may inform studies of biofilm formation and detachment in medical, environmental, or industrial contexts. This includes for instance, adhesion of biofilms on dental materials, water filtration systems, or microbial corrosion. Further exploration of a wider parameter space (flow rate, substrate composition, microbial traits) will be essential to refine our ability to predict microbial residence time on reactive substrates, and, in turn, better anticipate their feedbacks on surface reactivity across disciplines.

## Methods

### Aqueous solution and sample preparation

Solutions with two different saturation indexes with respect to calcite (*Ω* = 0.00 and *Ω* = 0.30, corresponding to far-from equilibrium conditions) were prepared to obtain more pronounced differences in bioweathering patterns^31^. The solutions were obtained by dissolving reagent grade NaCl (99.25 mM), NaHCO_3_ (1.02 mM) and CaCl_2_⋅2H_2_O (0 mM and 0.1425 mM, respectively) into deionized water before being filter-sterilized using 0.2 µm membranes to avoid external contamination and maintain axenic conditions throughout the duration of the experiments. The cyanobacterial strain *C. thermalis* PCC 7203, previously cultured in BG-11 medium under standard conditions until exponential growth phase, was centrifuged to form a cell pellet, which was then resuspended in a sterile aqueous solution saturated with respect to calcite and subsequently introduced into the fluid cell using a peristaltic pump. The pump was positioned upstream to actively drive the bacterial suspension through the reactor at a constant flow rate of 15 mL·h⁻¹ at the start of each experiment.

Calcite samples used in this study were cleaved along the natural {104} face from a transparent single crystal originating from the Massif des Écrins (Alps, France). The resulting calcite chips, each a few mm on a side and 1 mm in height, were then mechanically polished down to 0.25 µm by the Primeverre company (Montpellier, France). “Untreated” samples were used directly after polishing, while “pre-etched” samples were subsequently immersed in a 2 L tank of deionized water for ~15 h to induce surface pre-etching before starting the corresponding experiments, aiming to investigate calcite surfaces with different starting roughness.

### Experimental setup

The experimental set-up follows an approach similar to the one described in Ohmoto et al.^64^. In brief, calcite chips were placed with their mechanically-polished {104} surface facing up inside a see-through PEEK flow cell rigidly mounted on a VSI stage (Fig. S1a,b). A quartz sample of similar height was placed alongside the calcite surface to obtain a non-reactive reference surface during calcite dissolution and thus quantify calcite retreat rates (Fig. S1c). VSI measurements were performed in situ using a Zygo New View® 7300 Vertical Scanning Interferometer equipped with a glass-compensated objective, through a 100 µm-thick transparent glass at the top of the cell. During the measurements, the experimental solution was circulated through the fluid cell using a peristaltic pump placed upstream.

At the beginning of each experiment, the fluid cell was filled with a solution saturated with respect to calcite, in order to enable VSI imaging of the initial calcite surface in wet conditions, while preventing any dissolution. Cyanobacteria were then injected by temporarily connecting a conical 15 mL centrifuge tube containing the bacteria-enriched saturated solution. The experiments were conducted in air-conditioned room set at *T* = 20.0 ± 0.5 °C and pCO_2_ = 550 ± 100 ppm, with constant room illumination maintained throughout the experiments. Following the injection of the cyanobacterial suspension (which was flowed through the system for 1 min), the pump was temporarily stopped for ~2 min to facilitate bacterial attachment to the calcite surface. Subsequently, a fresh saturated solution at the desired *Ω* value was flushed through the fluid cell for ~5 min to remove non-adherent cells and ensure that only firmly attached cells remained.

Calcite dissolution was then initiated by flowing the appropriate aqueous solution—either at *Ω* = 0.00 or at *Ω* = 0.30—through the fluid cell at a constant rate of 10 mL·h⁻¹ for the entire duration of the experiments (~24 h). VSI topography images of pre-defined 400 × 400 µm^2^ regions of the calcite surface were acquired every 5 min throughout the experiment using a 10× objective and a 2x-zoom (lateral resolution, *dl* = 0.4 μm). A portion of the adjacent quartz surface placed next to the calcite chip was imaged within the same field of view to provide an inert reference unaffected by dissolution. Bacterial cells exhibited a distinct optical signature in the VSI height data: the reflected light from cell bodies, due to their refractive index contrast with the surrounding medium, was consistently interpreted as negative height anomalies relative to the calcite surface, appearing as shallow depressions, as previously reported by Waters et al.^65^. This property was leveraged here to localize individual cells across time, as described in the following subsection.

At the end of each experiment, residual surface-attached cells were removed using a 2% aqueous sodium dodecyl sulfate solution saturated with respect to calcite, following the same protocol used in a previous study^31^. Final surface topography images were acquired after removal of the bacteria, enabling microtopographic characterization of the calcite surface after dissolution in the absence of cellular coverage.

### VSI data processing

Time-resolved VSI datasets were processed to extract quantitative information on both the temporal dynamics of bacterial surface coverage and the evolution of the underlying calcite topography. To monitor cell coverage over time and estimate detachment rates, two approaches were employed. First, by leveraging the microtopography artifacts described previously, a thresholding approach was applied to the lower tail of the height histogram to isolate these features, enabling the reconstruction of dynamic surface exposure as cells detached. From the resulting binary masks, “bacteria residence time maps” were generated by summing binary masks over time. These maps provided a pixel-wise representation of how long each location remained covered by bacteria during the experiment. Additionally, bacterial coverage was also monitored via the grayscale optical images acquired simultaneously by the VSI system, where cells appeared darker than the surrounding calcite background, allowing for cell counting by contrast analysis. To quantify the fraction of dark regions within grayscale microscopy images, we developed a custom Python script leveraging adaptive thresholding. The input image was first converted to grayscale and processed using the *threshold_local* function from the Python library *scikit-image*, which computes a local threshold for each pixel based on the intensity statistics of its surrounding neighborhood. Binary segmentation was then performed, followed by morphological filtering to remove small artifacts. Connected components corresponding to dark clusters were identified and labeled. The cumulative area of these regions was computed and expressed as a fraction of the total image area, which was kept as close as possible from image to another (typically: ~300 µm × 100 µm). This method provides robustness against background inhomogeneity and internal intensity variation within dark aggregates. Finally, the surface area of dark regions was converted to a number of individual cells based on the diameter of individual cells (4 µm on average).

To assess whether bacterial presence influenced local dissolution, the final microtopography of the calcite surface was compared with the residence time maps through a pixel-wise correlation.

Lastly, to quantify net dissolution rates, the retreat of the calcite surface was measured relative to the adjacent inert quartz reference sample by tracking the relative height difference between the unreactive quartz and the reactive calcite over time, providing dissolution rates (i.e., surface retreat rates) expressed in terms of thickness of calcite removed per unit of time (Fig. S1c).

### Physiological tests

To verify that the cyanobacterial strain *C. thermalis* PCC 7203 remained viable under both saturation conditions (*Ω* = 0.00 and *Ω* = 0.30), confocal laser scanning microscopy (CLSM) was conducted on cells incubated for 72 h in batch reactors containing the same fluid compositions used in the in-situ experiments. CLSM imaging was conducted using a Zeiss LSM 710 microscope.

Additionally, to assess whether the bacteria remained metabolically active within the aqueous solutions employed in the experiments, pH evolution was monitored over time in parallel batch incubations under both saturation conditions (Fig. 5), providing a first-order approximation of the chemical microenvironment potentially developing at the cell–calcite interface as a result of photosynthetic processes.

## Supplementary information

Supplementary Information.

Supplementary Movie.

## Data Availability

The datasets generated during the current study are not publicly available due to their large size and complex structure, which would make them difficult to navigate without specific guidance. However, all data will be available from the corresponding author upon request.
