# Supplementary material for: In-situ laboratory monitoring of cyanobacterial influence on calcite dissolution
Source: Npj Mater Degrad. 2025 Nov 22;9(1):158. doi: 10.1038/s41529-025-00712-5 (PMC12727514; doi:10.1038/s41529-025-00712-5)
Supplement: Supplementary file 1 — Supplementary Information. [file 41529_2025_712_MOESM1_ESM.pdf]

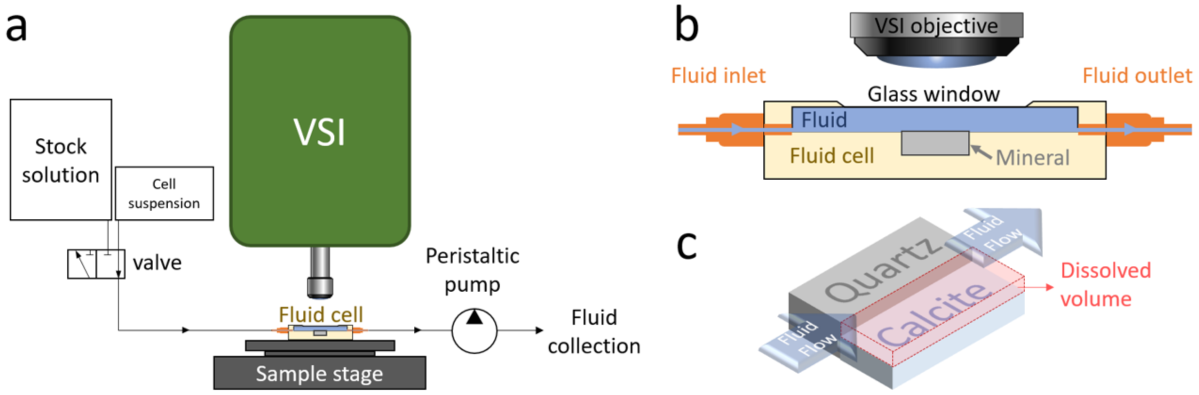

Figure S1. In situ flow-through VSI experimental set-up. (a) Schematic of the flow circuit: stock solution or cell suspension is directed via a valve to a see-through PEEK fluid cell mounted on the VSI stage; effluent is collected downstream of a peristaltic pump. (b) Cross-section of the fluid cell under the VSI objective showing the glass window, flowing solution, and the mounted mineral chip. (c) Plan view of the dual-substrate configuration with a calcite chip alongside a quartz reference; the red outline indicates the dissolved (retreat) volume quantified in situ by VSI.

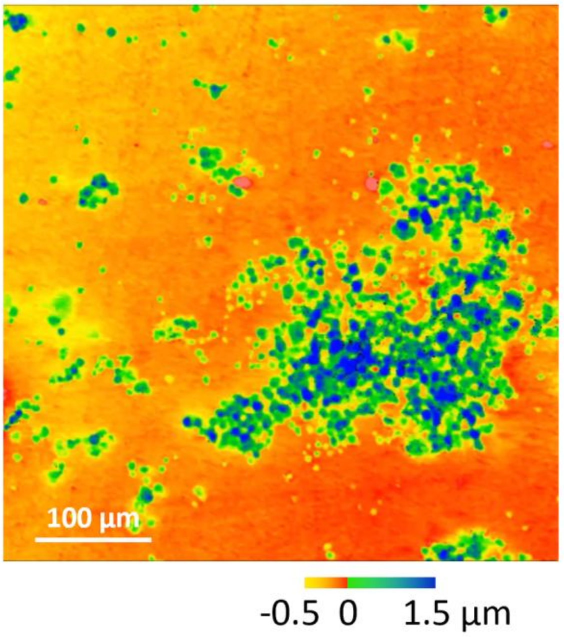

Figure S2. In situ time-lapse VSI imaging of bacterial attachment, detachment, and calcite surface evolution during dissolution. Representative VSI microtopography frame extracted from the time-lapse sequence showing an untreated calcite surface covered by *Chroococcidiopsis thermalis* cells (Experiment #1, $\Omega = 0.0$) under continuous flow. The full time-lapse sequence is provided as 'Supplementary Movie'.
